# Investigating the Relationship between Topology and Evolution in a Dynamic Nematode Odor Genetic Network

**DOI:** 10.1155/2012/548081

**Published:** 2012-09-28

**Authors:** David A. Fitzpatrick, Damien M. O'Halloran

**Affiliations:** ^1^Genome Evolution Laboratory, Department of Biology, National University of Ireland Maynooth, Maynooth, Co. Kildare, Ireland; ^2^Department of Biological Sciences, The George Washington University, 333 Lisner Hall, 2023 G Street NW, Washington, DC 20052, USA; ^3^Institute for Neuroscience, The George Washington University, 636 Ross Hall, 2300 I Street NW, Washington, DC 20037, USA

## Abstract

The relationship between biological network architectures and evolution is unclear. Within the phylum nematoda olfaction represents a critical survival tool. For nematodes, olfaction contributes to multiple processes including the finding of food, hosts, and reproductive partners, making developmental decisions, and evading predators. Here we examine a dynamic nematode odor genetic network to investigate how divergence, diversity, and contribution are shaped by network topology. Our findings describe connectivity frameworks and characteristics that correlate with molecular evolution and contribution across the olfactory network. Our data helps guide the development of a robust evolutionary description of the nematode odor network that may eventually aid in the prediction of interactive and functional qualities of novel nodes.

## 1. Introduction

For nematodes, olfaction is a central mode of survival. Olfaction contributes to the finding of food, hosts, reproductive partners, in the making of developmental decisions, and to the evasion from predators. Studies into the olfactory system of the model nematode *Caenorhabditis elegans* have yielded detailed descriptions of the molecular and cellular pathways that subserve the olfactory system [1–4]. These signaling pathways appear highly conserved across very divergent nematode species, and the sensory neurons have clear anatomical orthologs in distantly related nematodes [5]. Within the olfactory system of *C. elegans*, the ability to detect dilute volatile odors is mostly conferred by three pairs of neurons termed the Amphid Wing cells type A (AWA), Amphid Wing cells type B (AWB), and the Amphid Wing cells type C (AWC) [3, 4]. These cells are primary sensory neurons located within the sensory amphid organ of the head that forms part of an anatomically distinct subclass of amphid neurons in that they do not extend processes through the amphid opening, but instead their distal ciliated endings terminate inside a glial sheath cell [6]. Here we describe a composite odor genetic network in *C. elegans* that encompasses all three pairs of volatile odor-detecting neurons. We used this network to identify orthologous genes of the odor network in the nematode *Pristionchus pacificus*. We chose the nematode *P. pacificus* based upon three criteria: (1) it exhibits a similar lifestyle to that of *C. elegans, *in that they are both self-fertilizing hermaphrodites that will feed on bacteria, (2) conservation of the olfactory signaling pathway of *P. pacificus* to *C. elegans* has been validated experimentally [7, 8], and (3) because it is sufficiently divergent to generate meaningful divergence data (estimated divergence between *Caenorhabditis* and *Pristionchus* is between 280 and 430 million years ago [9, 10]). One of our goals in this study was to compare selective pressures across the nodes of the odor network by comparing substitutions at silent sites to that of nonsilent sites; this analysis is best done using more divergent sequences [11]. Using this nematode odor network we searched for relationships between pathway position with divergence, diversity, and contribution across the network. Then, we designed an interaction map of the network and investigated relationships between various metrics of interaction with molecular evolution, and contribution across the network. Our data is not a definitive map of odor signaling in *C. elegans* but represents a snap shot of current data, and by uncovering robust associations between network topology, evolution, and function we may ultimately design a framework that facilitates a level of predictive power over novel nodes within the network.

## 2. Materials and Methods

### 2.1. Sequences

*Pristionchus pacificus* orthologs were located by cross-referencing matches using the orthology databases: InParanoid_7 [12], OrthoMCL database [13], and the OMA orthology matrix Browser [14]. For each node in our network WormBase (version WS231) has defined a curated ortholog using WormBase-Compara [15]; however, we corroborated these predictions using reciprocal Blast [16] searches, and, by inferring relationships by reconstructing phylogenetic trees (see Figures S1–S7 in Supplementary Materials available online at doi:10.1155/2012/548081), we outline all the evidence for orthology in Table 1. Orthologs were aligned using the multiple sequence alignment software MUSCLE v3.6 [17], and gaps were systematically stripped from all sequences after alignment, and phylogenies inferred using PhyML [18]. To determine orthologs, WormBase-Compara uses the databases TreeFam [http://www.treefam.org/], InParanoid_7, KOGs [http://www.ncbi.nlm.nih.gov/COG/], OMA. The genes used in our network (Table 1), and their corresponding ortholog in *P. pacificus* (*WormBase* identifier for *P. pacificus *is denoted *Ppaxxx), *were *goa-1 *(G_o_ *α* subunit protein: *Ppa-goa1*);* egl-30 *(G_q_ *α* subunit protein: *Ppa-egl30*);* dgk-1 *(DGK*θ*, diacylglycerol kinase theta: *Ppa-dgk1*);* eat-4 *(vesicular glutamate transporter:* Ppa-eat4*);* egl-4/pkg-1 (*Protein Kinase G: *Ppa-pkg1*)*; tax-6 *(calcineurin type A: *Ppa-tax6*);* odr-1 *(receptor guanylyl cyclase:* Ppa17708*);* daf-11 *(receptor guanylyl cyclase:* Ppa-daf11*); *tax-2 *(cyclic nucleotide-gated channel *β* subunit: *Ppa-tax2*)*; tax-4 *(cyclic nucleotide-gated channel *α* subunit: *Ppa-tax4*)*; odr-3 *(G-protein *α* subunit: *Ppa-odr3*)*; gpa-3 *(G-protein *α* subunit: *Ppa-gpa3*)*; gpa-5* (G protein *α* subunit: *Ppa10789*)*; gpa-13 *(G-protein *α* subunit:* Ppa-gpa13*)*; arr-*1 (Arrestin: *Ppa-arr1*); *rgs-3* (regulator of G-protein signaling: *Ppa-rgs3*). To detect *C. elegans* versus *P. pacificus* 1 : 1 candidate orthologs for the randomization study we selected orthologs from the orthology database, InParanoid_7 [12]. We only included orthologs that are represented by 100% bootstrap support, and from this approach we obtain 5,666 1 : 1 orthologs. 

### 2.2. Analysis of Molecular Data

Synonymous (*d*_*S*_) and nonsynonymous (*d*_*N*_) substitution rates for orthologs were estimated using the methods of Yang and Nielsen [19] as implemented in yn00 in the PAML suite [20]. To test the null hypothesis that there is no above average selective pressure on these genes, we performed a randomization test where we determined the average *d*_*N*_/*d*_*S*_ value for 50,000 randomly assembled networks and compared with the average *d*_*N*_/*d*_*S*_ value for our odor network. Random networks were 16 genes in size and sampling permitted replacement. Measures of nucleotide diversity (*π*) were performed using DnaSP version 5 [21]. 

### 2.3. Network Analysis

Selection of vertices for the odor network was determined by mining literature databases. Network analysis was calculated using Cytoscape version 2.8.2 [22]. The network was treated as undirected and all network analyses available through Cytoscape version 2.8.2 were examined; these are average shortest path length, betweenness centrality, closeness centrality, clustering coefficient, degree, eccentricity, neighborhood connectivity, radiality, stress, and topological coefficient. The degree (*k*) of a node *n* is defined as the number of edges linked to *n*. The clustering coefficient (*C*_*n*_) reveals how connected the neighborhood of a node is by calculating the fraction of neighboring pairs, and for a node *n* it is defined as

(1) $$\begin{array}{c} C_{n}=\frac{2e_{n}}{k_{n}\left(k_{n}-1\right)}, \end{array}$$

where *k*_*n*_ is the degree of *n* and *e*_*n*_ is the number of connected pairs between all neighbors of *n*. Betweenness centrality (*C*_*b*_) is a measure of the fraction of shortest paths between node pairs (*s*, *t*) that pass through the node of interest, and for the node *n* it is calculated using the following formula:

(2) $$\begin{array}{c} C_{b}\left(n\right)=\sum_{s\neq n\neq t}^{}\frac{\sigma_{st}\left(n\right)}{\sigma_{st}}, \end{array}$$

where *s* and *t* are nodes different from *n*, *σ*_*st*_ denotes the number of shortest paths from *s* to *t*, and *σ*_*st*_(*n*) is the number of shortest paths from *s* to *t* on which *n* lies. 

 All chemotaxis indices (for both wildtype and mutant animals) were mined from previous publications (see references below). The chemotaxis index difference (C.I._diff_) for each mutant within each neuron (AWA, AWB, and AWC) was determined by calculating the difference between the wildtype chemotaxis index (C.I._wt_) and mutant chemotaxis index (C.I._mut_). For example, a mutant that presents an average AWA chemotaxis defect C.I. = 0.5, compared to the wildtype C.I. (C.I._wt-AWA_) of 0.9, would have a C.I._diff-AWA_ = 0.4 (i.e., subtract 0.5 from 0.9). In the case of long-term adaptation (LTA) mutants the C.I._diff_ was calculated by subtracting the C.I. value for LTA from C.I._wt_ of unadapted animals. Wildtype odortaxis: 0.9 (C.I._wt_ AWA); −0.95 (C.I._wt_ AWB); 0.85 (C.I._wt_ AWC) [4, 23]. Wildtype long-term adaptation: 0.5 (C.I._diff_ AWA); 0.65 (C.I._diff_ AWC) [24, 25]. *odr-3*: 0.6 (C.I._diff_ AWA); −0.7 (C.I._diff_ AWB); 0.5 (C.I._diff_ AWC) [23, 26]. *rgs-3*: 0.85 (C.I._diff_ AWC) [27]. *daf-11*: 0.6 (C.I._diff_ AWB); 0.5 (C.I._diff_ AWC) [28]. *dgk-1*: 0 (C.I._diff_ AWC) [29]. *eat-4*: 0.55 (C.I._diff_ AWC) [30]. *egl-30*: 0.65 (C.I._diff_ AWC) [29].*goa-1*: 0.6 (C.I._diff_ AWA); 0.6 (C.I._diff_ AWC) [29]. *odr-1*: 0 (C.I._diff_ AWA); 0.85 (C.I._diff_ AWC) [31, 32]. *tax-2*: 0.2 (C.I._diff_ AWA); −0.8 (C.I._diff_ AWB), 0.75 (C.I._diff_ AWC) [23, 33]. *tax-4*: 0.5 (C.I._diff_ AWA); 0.75 (C.I._diff_ AWC) [23, 34]. *egl-4*: 0.8 (C.I._diff_ AWA); 0.65 (C.I._diff_ AWC) [25, 34]. The phenotype index (PI) was then calculated as follows:

(3) $$\begin{array}{c} \text{P.I.}=\sqrt{{\left(\frac{{\text{C.I.}}_{\text{diff-mutx-AWA}}}{{\text{CI}}_{\text{wt-AWA}}}\right)}^{2}+{\left(\frac{{\text{C.I.}}_{\text{diff-mutx-AWB}}}{{\text{CI}}_{\text{wt-AWB}}}\right)}^{2}+{\left(\frac{{\text{C.I.}}_{\text{diff-mutx-AWC}}}{{\text{CI}}_{\text{wt-AWC}}}\right)}^{2}}. \end{array}$$

The PI for LTA mutants was measured by calculating the relative contribution of the LTA mutant to the adaptation response. For example, if the LTA response of wildtype animals normally decreases the C.I. value to 0.3 from 0.9, then the C.I._diff_ = 0.6; if the mutant is fully required (i.e., there is no LTA response at all in the mutant) for the LTA response, then the PI is calculated as follows: 0.6/0.6 = 1; which reflects the total contribution of the gene product to LTA in wildtype animals but that which is defective in the LTA mutant. The weighted phenotype index (WPI) was calculated for each group of mutants as follows:

(4) $$\begin{array}{c} \sum_{i=1}^{n}\frac{\left(\text{P.I.mu}{\text{t}}^{\text{1}}\right)\left(\text{wmu}{\text{t}}^{\text{1}}\right)}{\left(\text{wmu}{\text{t}}^{\text{1}}\right)}, \end{array}$$

where P.I.mut^*x*^ is the phenotype index (P.I.) for the mutant *x* (mut^*x*^), and wmut^*x*^ represents the number of odors for which mutant *x* (mut^*x*^) exhibits a mutant phenotype. 

## 3. Results

**Figure 1:**
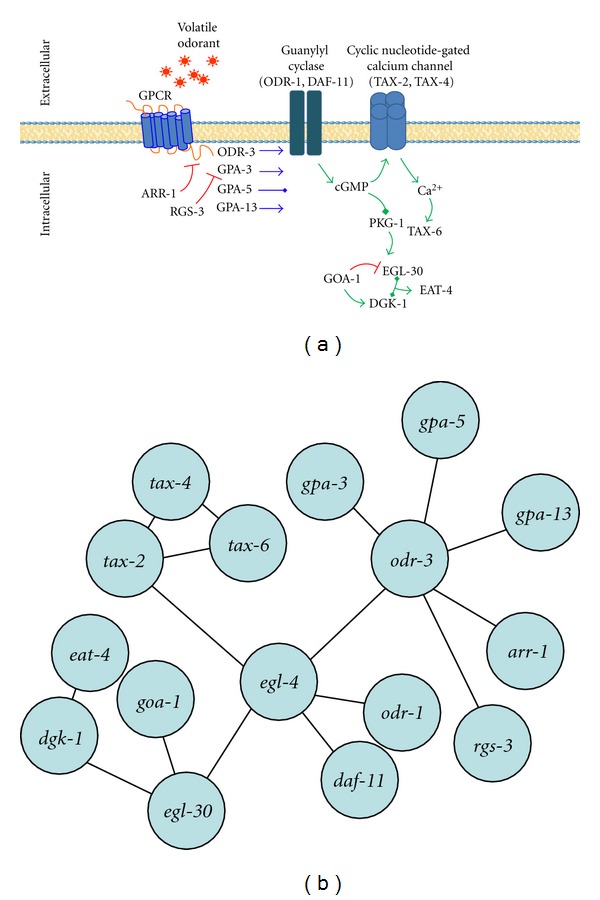
Odor-signaling pathway in *C. elegans* illustrated by pathway position and based on genetic interactions. (a) Summary of volatile odor signaling in *C. elegans*. (b) Schematic of an interaction-based odor network from *C. elegans*.

To compare divergence rate with pathway position in our network of dynamic odor transduction, we identified orthologous genes of the *C. elegans* odor network in *P. pacificus*, and divided the odor-signaling pathway members (Figure 1(a)) within this network into 3 categories: (I) regulators Class 1 (GPA-3 [35, 36], ODR-3 [26], GPA-5 [35, 36], GPA-13 [35, 36], ARR-1 [37] and RGS-3 [27]); (II) regulators Class 2 (EGL-4 [25], TAX-6 [38], ODR-1 [31], DAF-11 [28], TAX-2 [33], and TAX-4 [39]); (III) actuators (GOA-1 [40], EGL-30 [40], DGK-1 [40], and EAT-4 [41]). After binning pathway members into each category, we then calculated divergence within each group by comparing substitutions at silent sites to that of nonsilent sites and looked for patterns of correlation between topology and evolutionary rate. We did not observe a significant association between pathway position and divergence (pathway position: Spearman rank-order correlation coefficient *r*_*s*_ = 0.37, *P* = 0.15, Kendall's *τ* = 0.31, *P* = 0.13). We also designed an orthologous intragenus network using *Caenorhabditis japonica* and compared substitutions at silent sites to that of non-silent sites and looked for a pattern of correlation with pathway position. Using this intragenus network we also did not observe significant association between pathway position and divergence (pathway position: *r*_*s*_ = 0.28, *P* = 0.3, Kendall's *τ* = 0.17, *P* = 0.4), suggesting that the pathway position alone may not shape constraint in our odor network.

 Next, we designed an interaction map of the network and tested for correlations between divergence and various topological metrics (Figure 1(b)). The interaction-based odor network is a composite map of the pathways within the three pairs of neurons that subserve volatile odor recognition in *C. elegans* (these cells are the AWA, AWB, and AWC). The interaction network was designed based upon genetic interactions and localization studies. Edges may represent interactions from only one of the three odor sensing pairs of neurons, but placed in a composite framework of all three pairs of odor-sensing neurons to obtain an informative number of vertices. The network was treated as undirected. The network comprised 16 nodes with a network diameter of 5, a characteristic path length of 2.825, and an average number of neighbors equal to 2. For the interaction network we observed that a strong correlation between number of neighbors and average neighborhood connectivity (correlation = 0.857; coefficient of determination *R* ^2^ = 0.648). Plotting the number of neighbors against average clustering coefficient yields a strong pattern of correlation using a linear function (correlation = 0.868; coefficient of determination *R* ^2^ = 0.753); we observed that a decrease in clustering coefficient with an increase in number of neighbors as is typical of many networks. Network analysis was performed and the output of the analyses was tested for correlations with divergence by calculating the evolutionary rate for each node by comparing substitutions at silent sites to that of non-silent sites with the orthologous genes of the odor network in *P. pacificus*. We observed a significant association in two cases: (1) measures of betweenness centrality and (2) measures of degree. By comparing measures of betweenness or measures of degree with divergence we observed significant negative correlations (betweenness: *r*_*s*_ = −0.48, *P* = 0.02, Kendall's *τ* = −0.42, *P* = 0.03; degree: *r*_*s*_ = −0.57, *P* = 0.01, Kendall's *τ* = −0.44, *P* = 0.03). Comparing measures of degree to measures of betweenness we observed a strong correlation (correlation = 0.936; *R* ^2^ = 0.877); this is typical of networks containing nodes of high influence that increase their influence with increases in their number of edges and is not specific for our odor network. Then, we calculated molecular diversity across the interaction network and searched for patterns of association with metrics from the following characteristics of the network: pathway position, betweenness centrality, and degree. In each case we did not observe a significant pattern of correlation (betweenness: *r*_*s*_ = −0.35, *P* = 0.06, Kendall's *τ* = −0.3, *P* = 0.13; Pathway position: *r*_*s*_ = 0.4, *P* = 0.08, Kendall's *τ* = 0.37, *P* = 0.06; degree: *r*_*s*_ = −0.47, *P* = 0.06, Kendall's *τ* = −0.38, *P* = 0.06).

**Figure 2:**
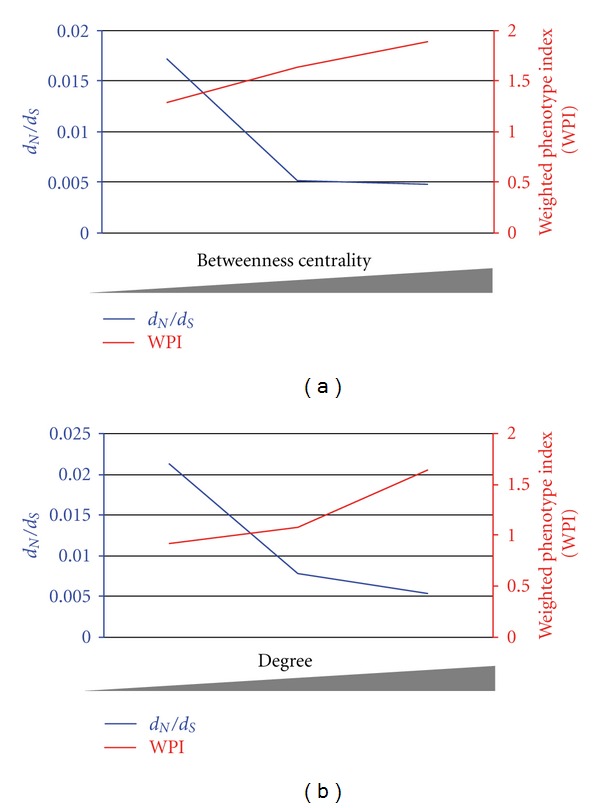
Plotting the relationship between divergence with measures of betweenness centrality or degree, and the relationship between contribution with measures of betweenness centrality or degree in a dynamic nematode odor network. (a) Divergence (left *y*-axis in blue) plotted against increasing measures of betweenness centrality (*x*-axis), and contribution (right *y*-axis in red) plotted against increasing measures of betweenness centrality. (b) Divergence (left *y*-axis in blue) plotted against increasing measures of degree (*x*-axis), and contribution (right *y*-axis in red) plotted against increasing measures of degree.

 Next, we developed a metric of contribution within the network we termed the weighted phenotype index (WPI), and compared the total contribution of each group with divergence when organized by pathway position, betweenness, or degree. In the case of pathway position we observed a weak correlation between divergence and contribution (*r* = 0.44), and in the case of betweenness or degree we observed strong negative correlations with contribution and divergence (Figure 2(a) betweenness: *r* = −0.92; Figure 2(b) degree: *r* = −0.76). We also examined the correlation between quantities of betweenness or degree for each node in our network with contribution, and again we observed robust correlations (Figure 2(a) betweenness: *r* = 0.9; Figure 2(b) degree: *r* = 0.79). From this analysis we uncovered patterns of association between measures of betweenness, and degree with evolutionary rate and contribution. 

**Figure 3:**
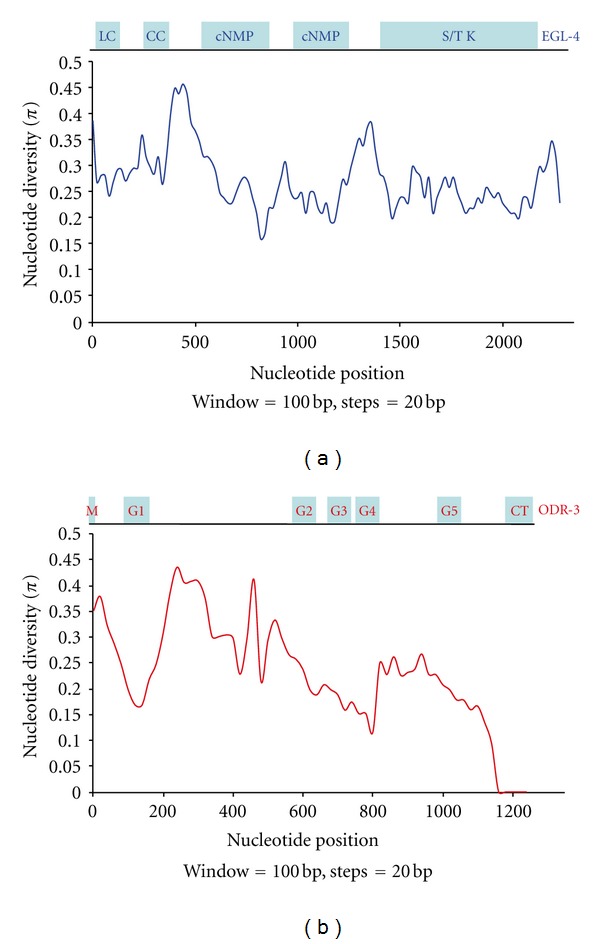
Sliding windows of nucleotide diversity within the coding regions of the genes *egl-4* and *odr-3*. Nucleotide position in the alignments (*x*-axis) is plotted against nucleotide diversity (*π*) on the *y*-axis. (a) Nucleotide diversity in *egl-4*. The shaded boxes on top of the graph illustrate the various functional domains within the EGL-4 protein. LC: low-complexity domain; CC: coiled-coil domain; cNMP: cyclic nucleotide-binding domain, S/TK: serine/threonine kinase domain. (b) Nucleotide diversity in *odr-3*. The shaded boxes on top of the graph illustrate the various functional domains within the ODR-3 protein. M: fatty-acid modification site; G1–G5: five alpha helices that comprise the GTPase domain; CT: C-terminal receptor interaction domain. In each case a 100-base-pair window was analyzed in 20-base-pair increments.

 The most highly connected nodes within our network are that of *odr-3* and *egl-4 *(also called* pkg-1*). ODR-3 is a G*α* subunit protein that transduces multiple stimuli within the network [26]. EGL-4 is a protein kinase G that facilitates both primary signal transduction as well as desensitization responses within the network [25, 34]. To examine how molecular diversity varies across these hub vertices, we performed a sliding window examination of the coding region of each gene using 100-base-pair windows in increments of 20 base pairs. In the case of *egl-4*, we observed only a few polymorphic peaks, with each peak associating with areas between domains of functional importance (Figure 3(a)); these are the N-terminal low-complexity domain (LC), the coiled-coil domain (CC), two cGMP-binding domains (cNMP), and the serine/threonine kinase domain (S/T K). In the case of *odr-3*, we observed more variability overall compared with that of *egl-4*, but again with most variable peaks associating with areas between domains of functional significance (Figure 3(b)); in particular, the five alpha helices (G1–G5) that comprise the GTPase domain, the receptor interaction C-terminal (CT) as well as the fatty acid modification site (M) (Figure 3(b)). We observed similar scales for nucleotide diversity for both *egl-4* (*π* = 0.27) and *odr-3* (*π* = 0.25) with each gene exhibiting polymorphic peaks in areas between domains of functional importance. The similar pattern of diversity is in keeping with similar trends of divergence between *egl-4* and *odr-3*: *egl-4 d*_*N*_/*d*_*S*_ = 0.0041, *d*_*N*_ = 0.1183, *d*_*S*_ = 29.1777; *odr-3 d*_*N*_/*d*_*S*_ = 0.0054, *d*_*N*_ = 0.1132, and *d*_*S*_ = 21.1204. 

**Figure 4:**
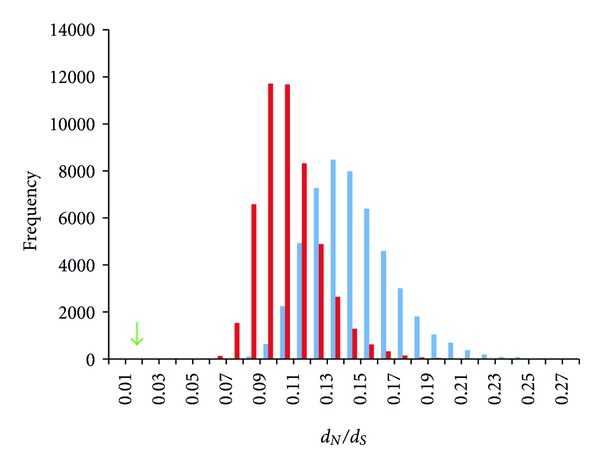
Frequency distribution of average *d*_*N*_/*d*_*S*_ values from 50,000 randomization sets comprising 16 node groupings from 5,666 1 : 1 orthologs between *C. elegans* and *P. pacificus* (blue bars). Red bars indicate frequency distribution of average *d*_*N*_/*d*_*S*_ values from 50,000 randomization sets of 16 node groupings that include 6 G*α* subunit proteins in each; this control group was examined to investigate potential bias in our network by having 6 G*α* subunit proteins within our odor genetic network. The *x*-axis represents binned divergence categories plotted against the frequency on the *y*-axis. The green arrow indicates the actual average *d*_*N*_/*d*_*S*_ value of the nematode odor network.

 Overall, nodes within the odor network are undergoing purifying selection, with an average *d*_*N*_/*d*_*S*_ value across the network of 0.012 for *P. pacificus* versus *C. elegans*. To place this value in a context of global divergence rates between *C. elegans* and *P. pacificus* we examined divergence across 5,666 pairs of 1 : 1 orthologs, then generated randomized data sets (50,000 in total) comprising an equal number of nodes as our odor network (i.e., 16), and examined the frequency of mean *d*_*N*_/*d*_*S*_ values in each case (Figure 4, blue bars). From this analysis we found an average *d*_*N*_/*d*_*S*_ value = 0.14 which is more than an order of magnitude higher (~11.6X) than what we observed for our dynamic odor network. To control for the number of G*α* subunit proteins within our odor network we also conducted a randomization trial whereby *d*_*N*_/*d*_*S*_ averages from 50,000 random data sets of 16 nodes that contain 6 G*α* subunit genes in each case were generated (Figure 4, red bars). In this control experiment we still observed a significantly higher average *d*_*N*_/*d*_*S*_ value (*d*_*N*_/*d*_*S*_ average = 0.106) than for our odor network. Taken together, this suggests that the dynamic odor-signaling network is shaped by a more pronounced purifying selection than what guides global constraint across the genome. 

## 4. Discussion 

Positional rate variation (PRV) has been tested within numerous biosynthetic pathways where it has been demonstrated that upstream genes (perhaps on account of greater pleiotropy) undergo more selective constraint than downstream genes [42–44]. However, it is unlikely that this effect holds true for all signaling pathways as input and network architecture will vary widely. In the case of sensory signaling pathways that subserve an environment versus perception “arms race,” the idea of upstream genes exhibiting greater diversity may be more applicable. This trend was observed in a study of the *Drosophila* innate immune system where the authors demonstrated that recognition genes undergo higher levels of positive selection than immune effector genes [45]. In our network we did not observe a significant correlation between pathway position and divergence, which was in keeping with previous observations from data on intragenus networks of odor signaling in *Caenorhabditis* [46]. However, by comparing measures of degree or centrality with divergence, we did observe significant negative correlations. This may suggest that pathway position is not the only factor in shaping constraint or change in our network, but rather network characteristics such as connectivity may play major roles in guiding molecular evolution. Negative correlations between divergence with node relevance, and positive correlations between essentiality with node relevance have been reported previously and may represent a general trend of many biological networks [47, 48]. 

 By comparing global levels of divergence between *C. elegans* and *P. pacificus* we found a pervasive theme of constraint across the genome; however, through our randomization study (Figure 4) we found that global levels of constraint are an order of magnitude lower than the level of constraint across the odor network. This suggests that the odor-signaling pathway represents a fixed circuit whose network properties are preserved by strong purifying selection. Low levels of divergence have been reported within *Caenorhabditis* for members of the olfactory pathway [46], and previously we have found that a large component in Regulators Class 1 and 2 has undergone extensive nematode-specific gene duplication events, namely, the G*α* subunit proteins and guanylyl cyclase proteins [49, 50]. Many of these genes are either exclusively or highly expressed in primary sensory neurons [26, 28, 31, 35, 36, 51]. This genetic expansion facilitates multiple capacities that shape developmental and survival strategies through intercellular and intracellular processing of polymodal sensory input. Many of these duplicates are present in all four major clades of the phylum nematoda [49] suggesting that the odor-signaling network arose early in nematode evolution and has undergone neofunctionalization events that are preserved by strong functional constraint. 

## Supplementary Material

The Supplementary information file contains Figures of relationships inferred by reconstructing phylogenetic trees for seven families of genes within our odor signaling network. These are: 1) guanylyl cyclases; 2) diacylglycerol kinases; 3) vesicular glutamate transporters; 4) G protein alpha subunits; 5) protein kinase G; 6) calcineurin; and 7) cyclic nucleotide gated channel subunits.

## Figures and Tables

**Table 1:** Orthology support for each node within the odor-signaling network.

*C. elegans* gene	*P. pacificus* ortholog	Evidence of orthology
*goa-1*	PPA23648/*Ppa-goa-1 *	WormBase-Compara
*odr-3*	PPA14189/*Ppa-odr-3 *	InParanoid; OMA; WormBase-Compara
*egl-30*	PPA31690/*Ppa-egl-30 *	WormBase-Compara
*gpa-3*	PPA09867/*Ppa-gpa-3 *	InParanoid; OMA; WormBase-Compara
*gpa-5*	PPA10789	OMA; WormBase-Compara
*gpa-13*	PPA31402/*Ppa-gpa-13 *	WormBase-Compara
*eat-4*	PPA15025/*Ppa-eat-4 *	InParanoid; WormBase-Compara
*dgk-1*	PPA00312/*Ppa-dgk-1 *	InParanoid; OMA; WormBase-Compara
*odr-1*	PPA17708	InParanoid; OMA
*daf-11*	PPA14907/*Ppa-daf-11 *	WormBase-Compara
*tax-6*	PPA09320/*Ppa-tax-6 *	WormBase-Compara
*pkg-1*	PPA27475/*Ppa-pkg-1 *	InParanoid; OMA; WormBase-Compara
*tax-2*	PPA07436/*Ppa-tax-2 *	InParanoid; OMA; WormBase-Compara
*tax-4*	PPA02388/*Ppa-tax-4 *	InParanoid; OMA; WormBase-Compara
^1^ *rgs-3*	PPA01970/*Ppa-rgs-3 *	InParanoid; OMA; WormBase-Compara
^1^ *arr-1*	PPA21763/*Ppa-arr-1 *	WormBase-Compara

^1^Relationship support was confirmed by reconstructing phylogenetic trees for each gene except *rgs-3* and *arr-1* as they represent single-gene families, and therefore not possible to reconstruct phylogeny (see Figures S1–S7).
