# Supplementary figures and images for: Investigating the Relationship between Topology and Evolution in a Dynamic Nematode Odor Genetic Network
Source: Int J Evol Biol. 2012 Sep 28;2012:548081. doi: 10.1155/2012/548081 (PMC3465961; doi:10.1155/2012/548081)

## Slide 1
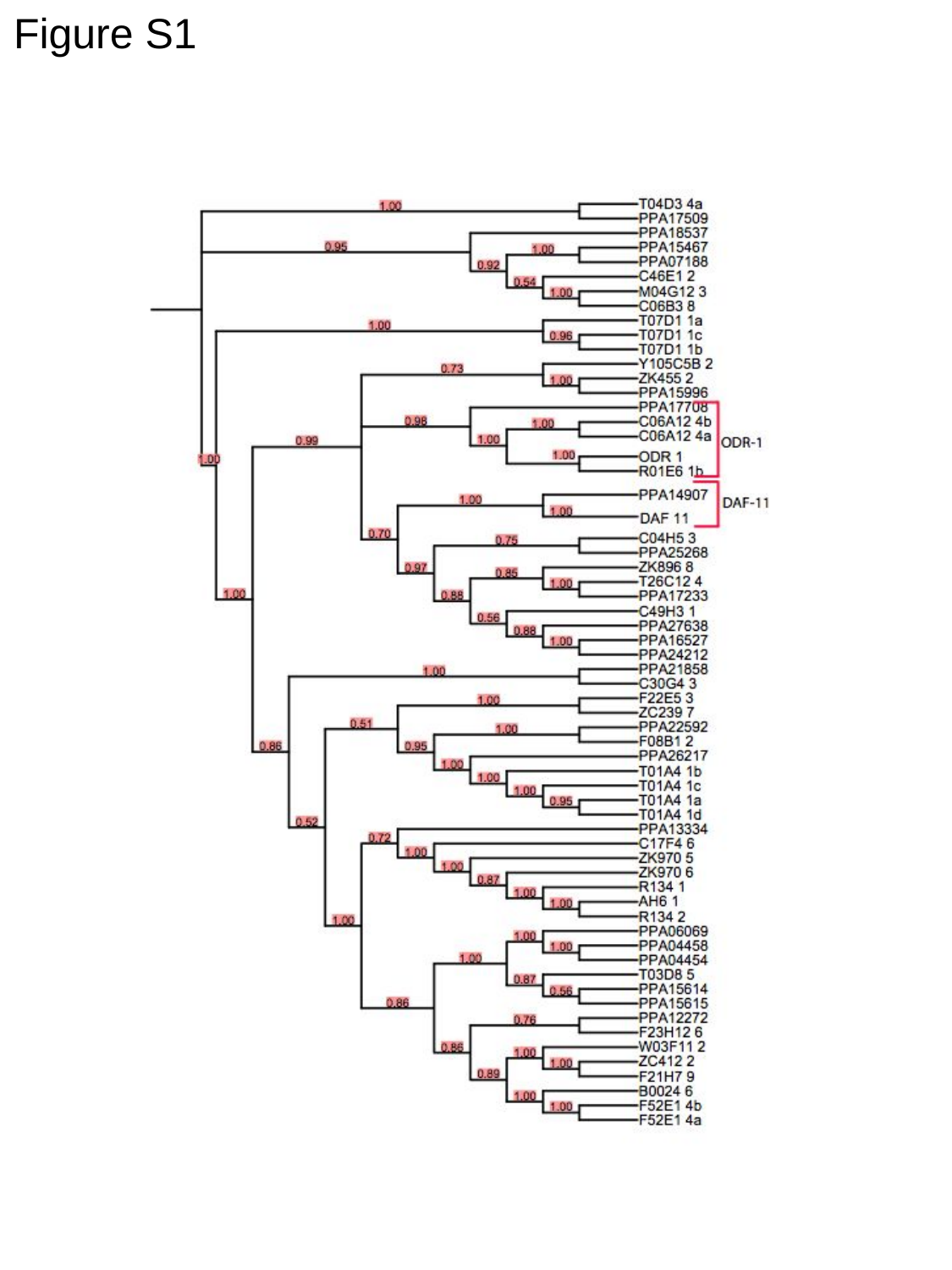

Figure S1

Supplement: Supplementary file 1 — The Supplementary information file contains Figures of relationships inferred by reconstructing phylogenetic trees for seven families of genes within our odor signaling network. These are: 1) guanylyl cyclases; 2) diacylglycerol kinases; 3) vesicular glutamate transporters; 4) G protein alpha subunits; 5) protein kinase G; 6) calcineurin; and 7) cyclic nucleotide gated channel subunits. [file 548081.f1.ppt]

## Slide 1
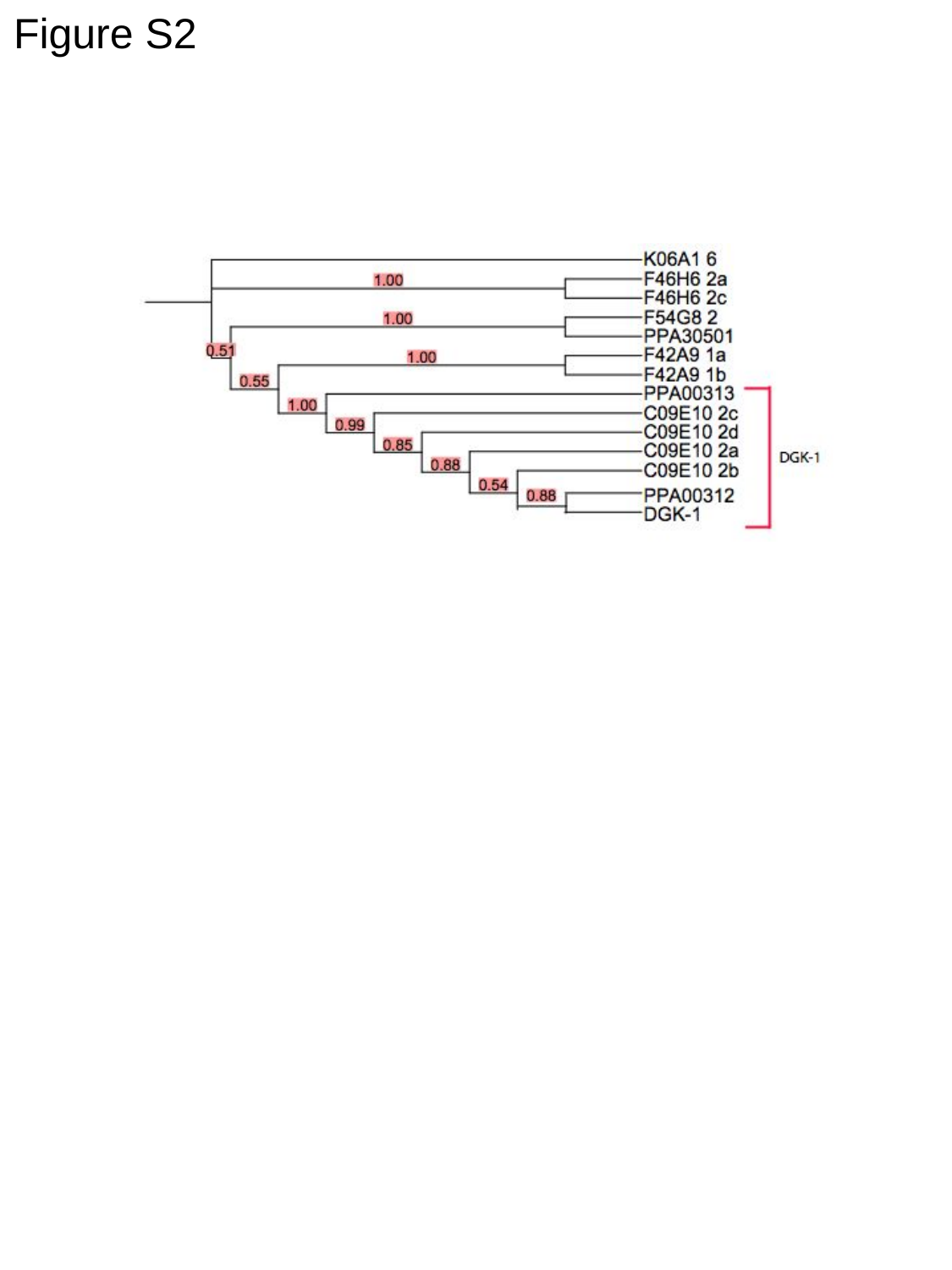

Figure S2

Supplement: Supplementary file 2 [file 548081.f2.ppt]

## Slide 1
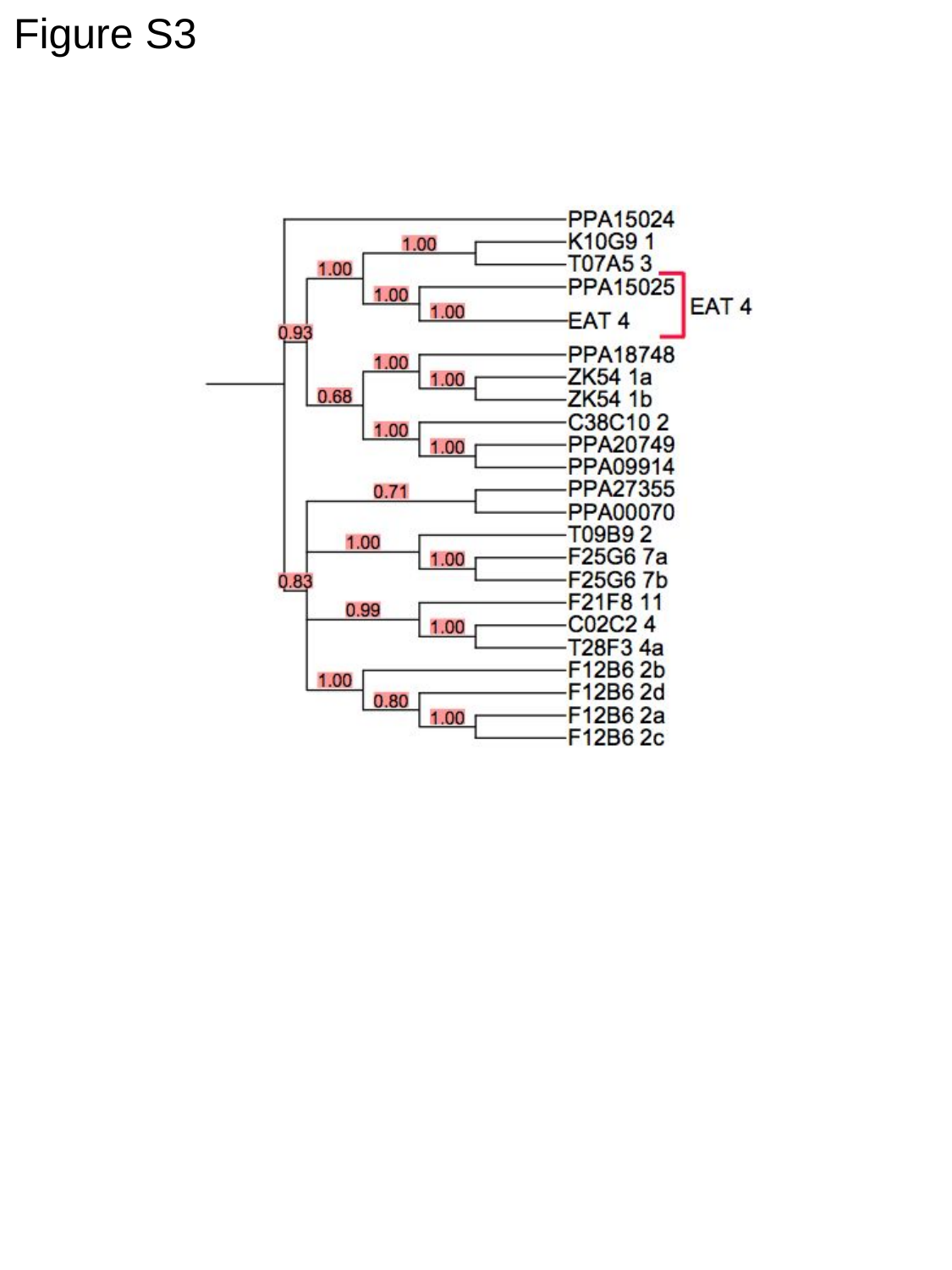

Figure S3

Supplement: Supplementary file 3 [file 548081.f3.ppt]

## Slide 1
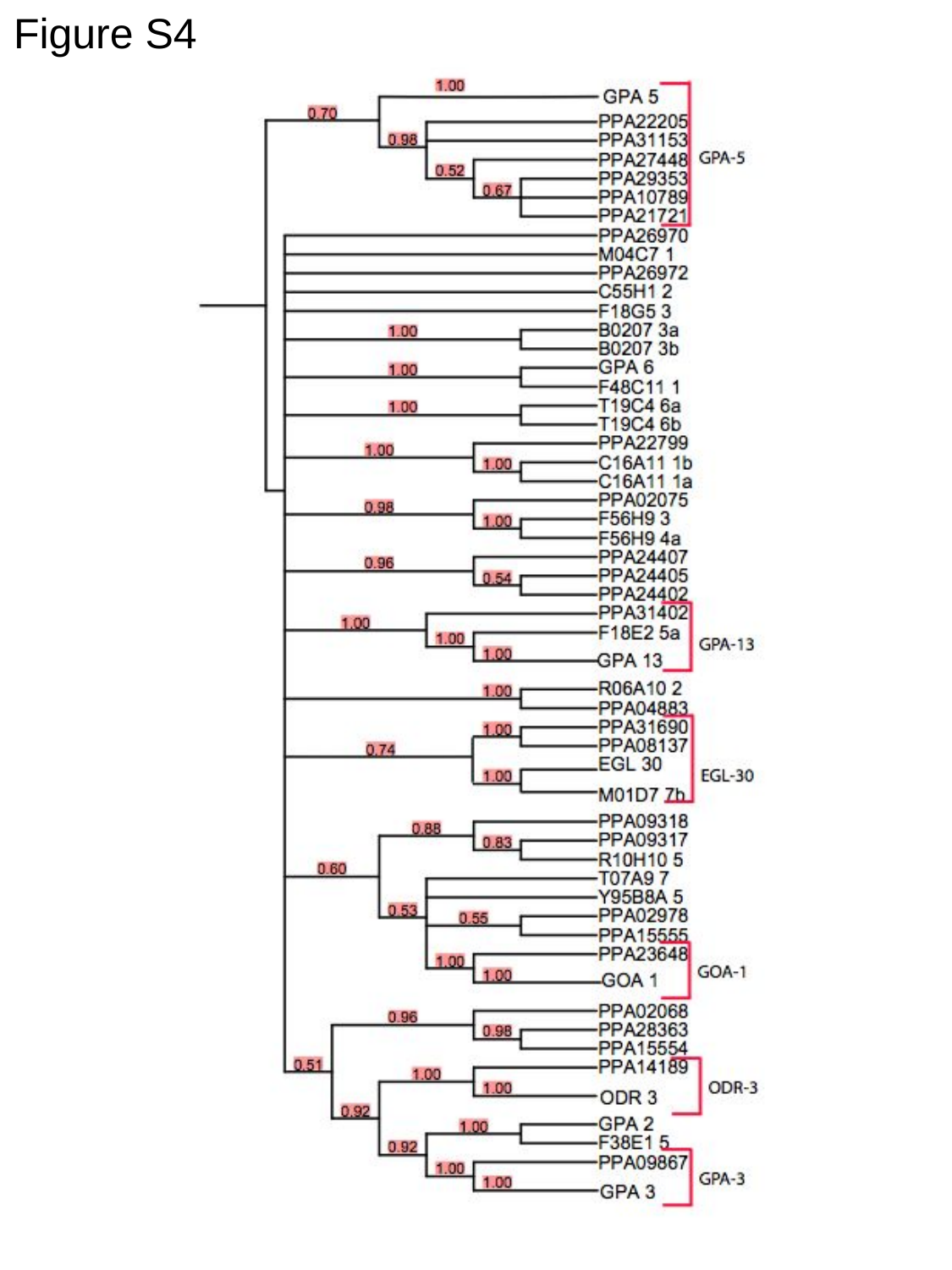

Figure S4

Supplement: Supplementary file 4 [file 548081.f4.ppt]

## Slide 1
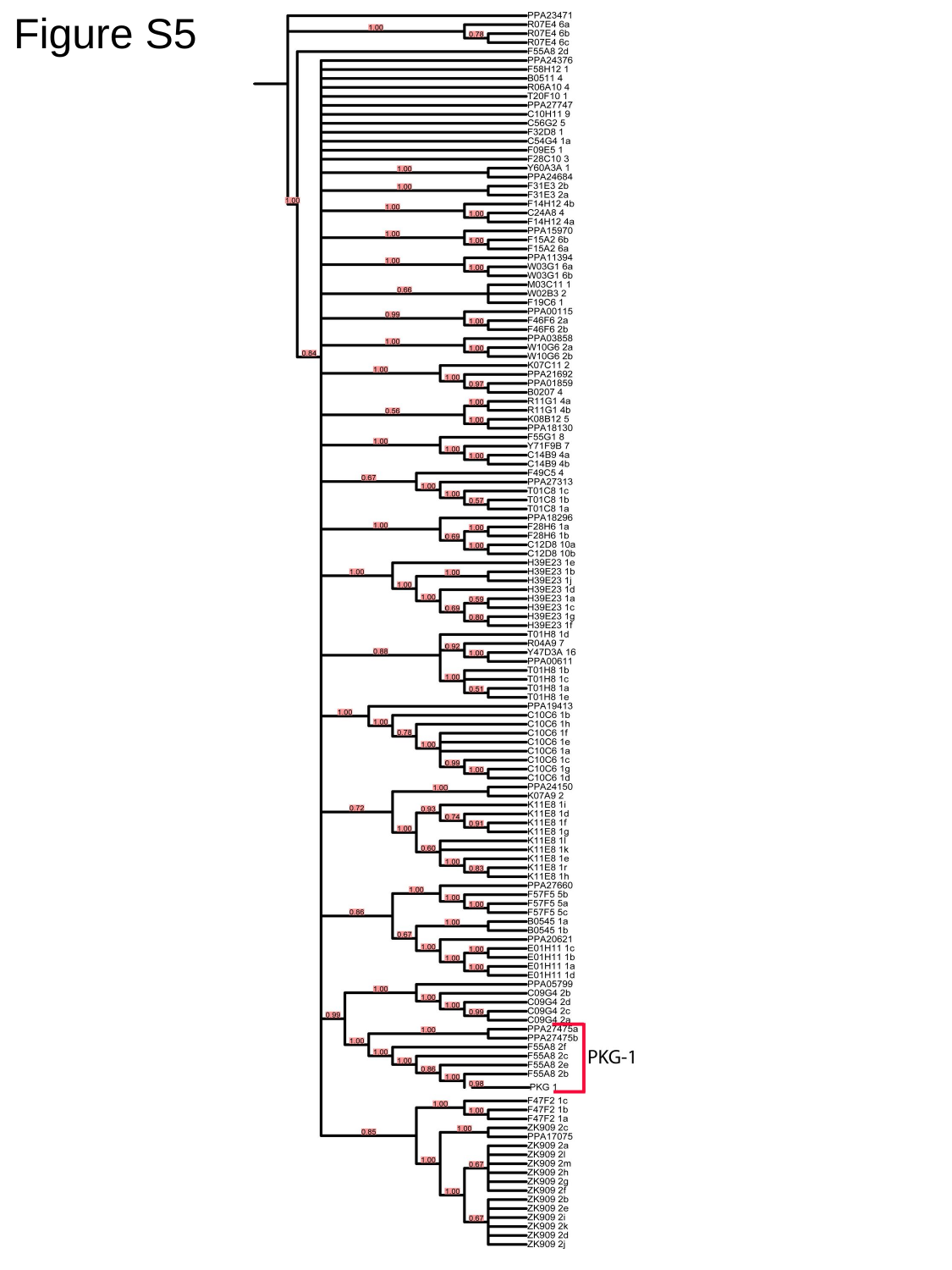

Figure S5

Supplement: Supplementary file 5 [file 548081.f5.ppt]

## Slide 1
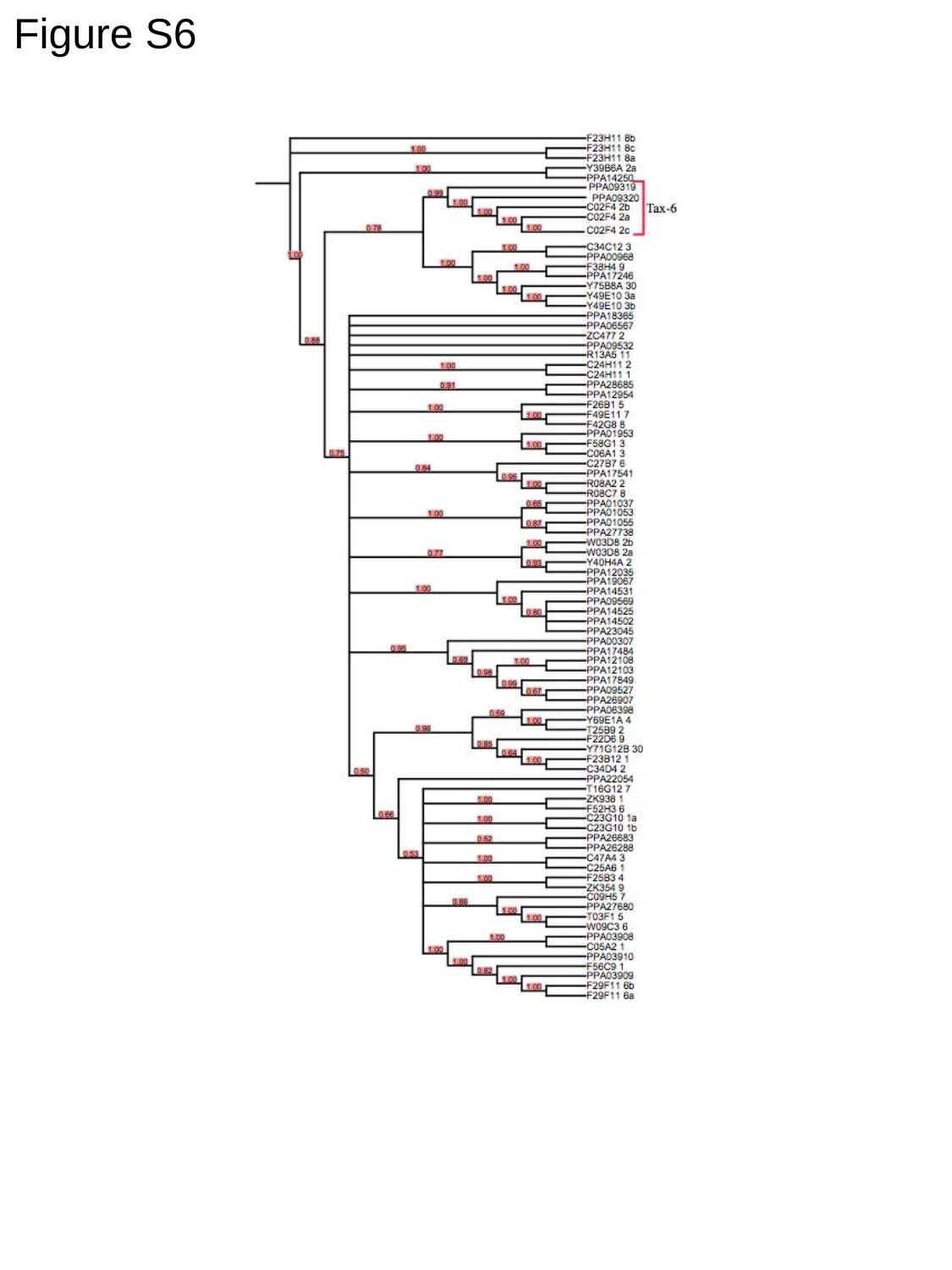

Figure S6

Supplement: Supplementary file 6 [file 548081.f6.ppt]

## Slide 1
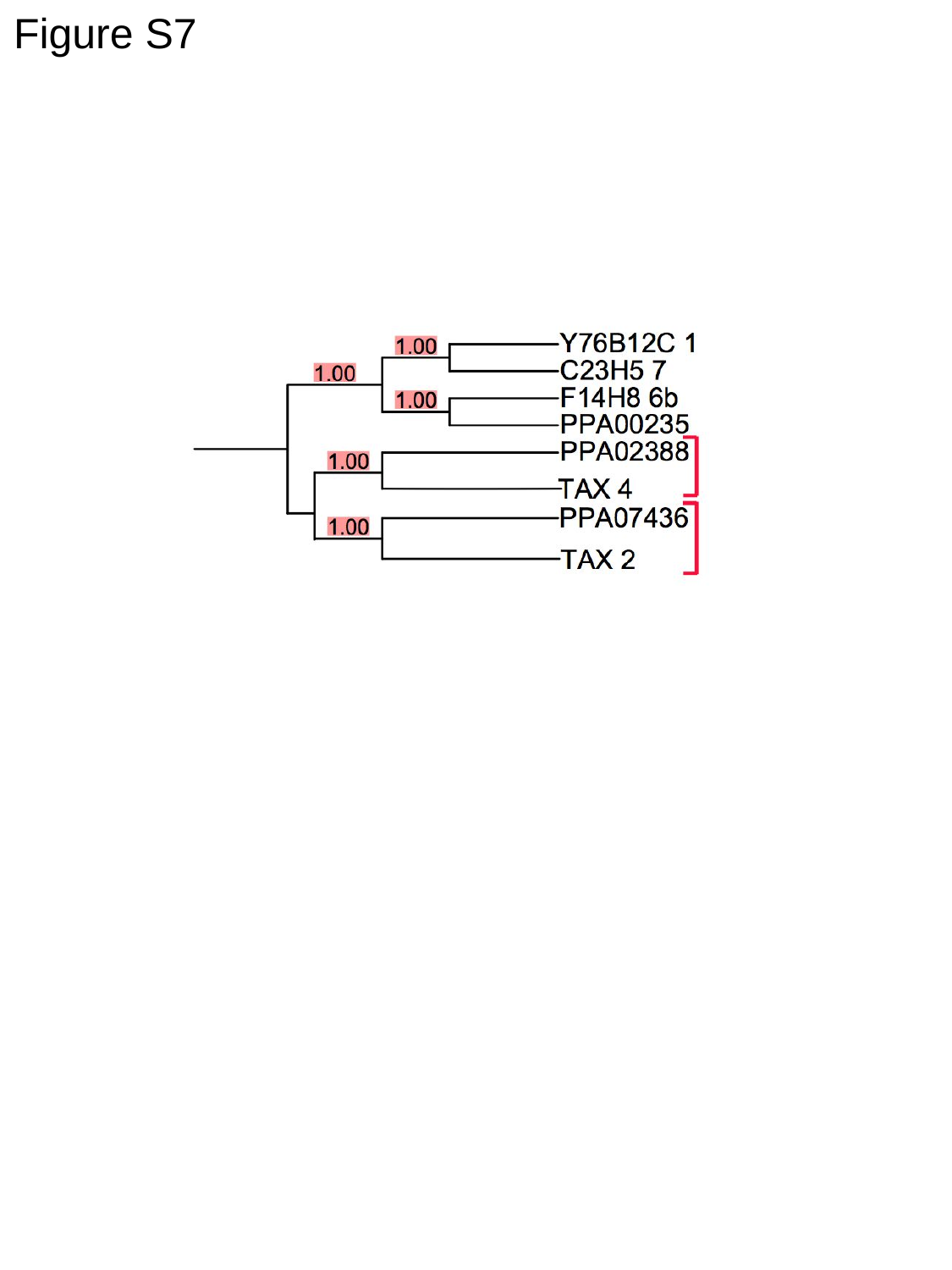

Figure S7

Supplement: Supplementary file 7 [file 548081.f7.ppt]
